# Preparation and *in-vitro* Antibacterial Evaluation of Electroless Silver Coated Polymers 

**Published:** 2010

**Authors:** Mohammad Reza Fazeli, Vahid Hosseini, Fazel Shamsa, Hossein Jamalifar

**Affiliations:** a *Department of Drug and Food Control, School of Pharmacy and Pharmaceutical Sciences Research Center, Tehran University of Medical Sciences, Tehran, Iran.*; b *Department of Pharmaceutical Chemistry, School of Pharmacy and Pharmaceutical Sciences Research Center, Tehran University of Medical Sciences, Tehran, Iran.*

**Keywords:** Electroless plating, Polymer, Indwelling catheters, Bacterial adhesion, Silver coated

## Abstract

Long-term use of indwelling medical catheters has often been hindered by catheter-associated nosocomial infections. In this study the effectiveness of silver coating of polystyrene and polyethylene polymers was investigated. Polymer pieces of 2 cm^2^ each were coated with a thin layer of silver using electroless plating technique. Silver-coated polymers were challenged with cultures of four different microorganisms known for their involvement in nosocomial infections in both solid and broth media. The tested bacteria included *Staphylococcus aureus, Staphylococcus epidermidis, Escherichia coli *and *Pseudomonas aeruginosa*. Silver release from the coated polymers was 2-5 μg/cm^2^ which was confirmed by chemical and biological methods. The silver coating thickness ranged between 20-450 nm. *P. aeruginosa *and *S. aureus *were the most adherent bacteria to polystyrene sheets while *E. coli *showed minimum adherence effect. The survival rate of different bacteria after 80 min in a time course experiment tended to dominate *E. coli *as the most sensitive bacteria to the effect of silver with zero survival rate while around 4% of *P. aeruginosa *were detected after same period. Silver coating of indwelling polymers by electroless technique seems promising in combating nosocomial infections due to long-term catheterization.

## Introduction

Indwelling urethral catheters (IUC) are disposable systems consisting of catheters, tubing and drainage bags, which are used in about 15-25% of all hospitalized patients (1). Urinary tract infections (UTI) are one of the most common nosocomial infections (2) accountable for approximately 40% of all hospital acquired infections, and 80% of these are associated with the use of urinary catheters (3). Multi-centre studies of intensive care units found the prevalence of UTI, to be 1-6% of all identified infections (4, 5). Catheter associated urinary tract infections would prolong the mean length of hospital stays by 2.4 to 4.5 days (6, 7), and may increase in-hospital mortality (8). Therefore, the best management of patients with IUC is the prevention of the infectious complications.

Although daily decontamination of drainage bags with a dilute sodium hypochlorite solution allowed usage of the same bag without increased risk of bacterial contamination (9), daily flushing of the catheter with normal saline, chlorhexidine or hydrogen peroxide offers no benefits in terms of reduced bacteriuria or catheter obstructions (10-13).

Approximately 5 millions central venus catheters (CVCs) are inserted annually in the USA and more than 200,000 catheters related bloodstream infections are reported each year (14). Microbial biofilms might be formed after 24 h of catheter insertion and are very resistance to antibiotics and other antimicrobial agents (15). Different approaches are practiced to achieve catheters with antimicrobial properties. For instance, ciprofloxacin or metallic particles have been absorbed on the surface of polymers (16-18). It has been reported that silver or silver oxide provides protection against UTI in high risk patients (19-21). Silver has long been a candidate for its oligodynamic effects. Silver nitrate has been used as traditional antiseptic agent, because silver ions attach to the cytoplasmic proteins of bacteria and denature them. The ions are then dissociated from the proteins and will act as free agents following bacterial death. This behavior defines the oligodynamic action of silver showing antibacterial effect at tiny concentrations (22, 23).

Some physical and chemical methods such as PVD (physical vapor deposition), CVD (chemical vapor deposition) and ion spattering could be used for producing silver alloy metallic layer. In this paper the wet chemical deposition process (electroless plating) for forming the laminar-alloy of silver was used, and its effect on some microorganisms was investigated. In addition a method for detection and quantitative analysis of silver ions released from the coating was also presented.

## Experimental

*Materials*

The microorganisms used in this study included *Staphylococcus aureus *ATCC 6538p, *Pseudomonas aeruginosa *ATCC 9027, *Staphylococcus epidermidis *ATCC 14990, and *Escherichia coli *ATCC 8739 all were stocks of the Department of Drug and Food Control, School of Pharmacy, Tehran University of Medical Sciences. The bacterial culture media included nutrient broth (NB) and soybean casein digest broth (SCDB) purchased from Merck Co. (Germany).

Latex Foley urinary catheters and nelaton urinary catheter were purchased from Kawamoto Company (Malaysia). Polystyrene and polyethylene sheets from Jamposhine Co. (Malysia) were used. Also variety of nylon stitch string (Soha Helal Co., Iran) was tested.

Sulfuric acid, nitric acid, chromic oxide, silver nitrate, dithizone, NaOH, sodium hypochlorite, tin chloride, CHCl_3_, CCl4, and gold chloride were from Merck Co. (Germany). Tecna 6, Tecno-GAZ Ultrasonic was used as cleaning bath (Italy). 

*Preparation of silver coated polymers*

Initially, the polymers were degreased by trade alkaline detergent followed by immersion washing with tap water. The latex Foley catheter polymer was then etched with sodium hypochlorite solution (5% w/v) at ambient temperature for 5 min. The nylon and polystyrene polymers were etched with solution of NaOH (20% w/v) at ambient temperature for 15 min. Chromic-sulfuric acid (300 g/L) was applied to etch the polyethylene polymer at 65 °C for 5 min. Sensitizing the polymers was achieved by tin chloride solution (5% w/v) at pH 2.5 in room temperature for 15 min (24). Pieces of polymers were then rinsed with deionized water, immersed in electroless silver bath with 5 A° per second deposition rate which deposits a nanometric pure silver layer over the activated polymers. The deposition time applied varied between 2 to 15 min. After one step rinsing with deionized water and immersion plating in stabilizing solution containing gold chloride (0.06 g/L) in ambient temperature, the polymeric pieces were rinsed with double distilled water and dried under warm air. 

*Quantitative estimation of silver ions released from the polymers using dithizone*

*Preparation of pure dithizone indicator*

Dithizone is purified by filtering its saturated solution in carbon tetrachloride or chloroform. The filtered dithizone solution was transferred to a separatory funnel, followed by ammonia solution (1-2%) and the mixture was stirred. The organic layer was separated and added to an aqueous solution in two 5 mL portions, shaking the content each time with subsequent separating the organic layer. The aqueous solution was made acidic by sulfuric acid (5%) followed by few drops in excess. Dithizone is liberated as blackish violet flakes extractable by chloroform (25).

*Silver-dithizone spectrum*

1 to 5 μg of silver ion was added to 3 mL of dithizone solution in chloroform (1x10^-5^), and mixed well. The absorption spectrum of the chloroform layer showed maxima at 460 and 605 nm. 

*Silver ion release detection*

One mL of the solution exposed with coated polymer was treated with 3 mL of indicator solution (dithizone, 1 x 10^-5^ M) and mixed well. Absorption peaks of its chloroformic layer were taken as a mean for the presence and quantification of silver ions released from the polymer using the above calibrated range.

*Microbial analysis*

*Antimicrobial analysis in liquid media*

Test tubes each containing 6 mL of nutrient broth were sterilized by autoclaving at 121°C. Three pieces of 2 cm^2^ coated polymers were immersed in the NB tubes to achieve a 1:1 (S^2^/V) of coated surface to culture volume. Non-coated polymers were considered negative control. The tubes were incubated with 20 μl of an over night culture of the test strains followed by incubation at 37 °C for 24 h (26). 

*Antimicrobial analysis in solid media*

SCDB agar plates (10 cm diameters) were surface cultured using swabs soaked in different bacterial cultures. Pieces of the coated catheters and plates made of latex, nylon and polystyrene were placed on the surface of agar and were left for 3 h at 4°C followed by incubation at 37°C for 24.

*Bacterial adhesion assay*

Bacterial suspensions equivalent to 0.5 McFarland of individual bacteria were prepared. Coated polystyrene sheet pieces (2 cm^2^) were immersed in bacterial suspension for 1 h. Samples were taken at 0, 40 and 80 min intervals and washed twice with sterile distilled water. The bacterial treated pieces were then placed in tube containing 2 mL of normal saline and were placed in ultrasonic cleaner bath at 37°C for 3 min. The contents were mixed well, followed by culturing on SCDB plate and incubated at 37°C for 24 h (26).

*Determination of silver ion release from polymer surface into microbial culture*

Blank round polystyrene sheet discs with 0.5 cm diameter were soaked in solutions containing different concentrations of silver ions (0.5, 1, 2, 5, 10 and 20 μg/mL). The discs were dried in a desiccator before loading on SCDB plates swabbed with *P. aeruginosa *as stated in section bacterial adhesion assay. The inhibition zones around the discs were investigated following 24 h incubation at 37°C. Silver ions released by the polymers were estimated by measuring the inhibition zones around the coated polymers compared to those of silver loaded discs. 

*Coating thickness and plating rate tests*

After pre-treating of a very smooth and glance surface such as glass (2 cm × 2 cm) they were plated as stated in section 1.2. The coated glasses were soaked in 65% w/v nitric acid after coating process was fulfilled. The acid then neutralized and diluted 1 to 100 with double distilled water. A 1 mL sample was taken from this solution and analyzed using dithizone indicator. The weight of the dissolved silver ions computed as stated in section microbial analysis. The thickness of the layer was calculated using the following equation:

P = M/V → V = A × d = M/P → d (n) = [M (g) / (P (g/m^3^ ) × A (cm^2^))] x 10^7^

Where M, P and A are silver weight, density and the plate surface area, respectively. The thickness obtained from this formula expressed by nanometer.

The plating rate could be calculated by dividing the layer thickness by plating time. 

## Results and discussion

Various techniques have been used for silver coating of some medical devices, such as intra uterine catheters. These methods include silver reducing by electrical current (27), silver coating by physical or chemical evaporation (28), chemical vapor deposition of silver coating on polymeric substrates (29). Although the silver surface produced by electricity is very pure, adjusting its thickness is somehow difficult (27). In the chemical and physical evaporation methods, high temperature and low pressure are used. In this method, although the deposition on conducting and non-conducting materials is possible, using spatial reactors and the difficulties in the metal deposition in narrow and long substrates has restricted its application to catheters (28). Therefore, in this study the electroless method (24) has been applied for deposition of silver ions on some polymeric substrates and subsequent evaluating the deposition by chemical and microbial methods.

*Microbial section*

**Figure 1:**
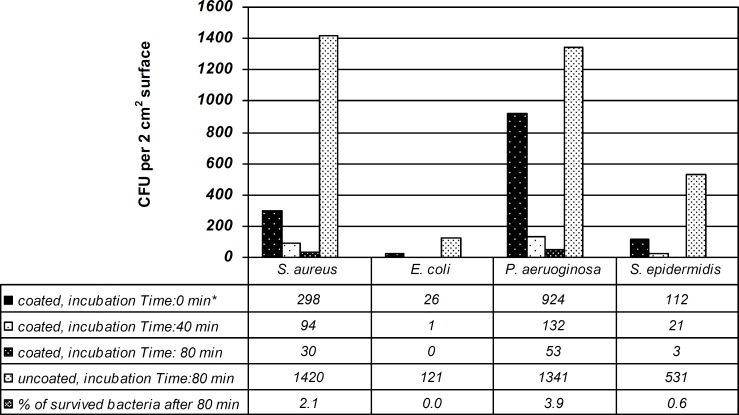
Time course of bacterial adherence to silver coated polystyrene sheets (* The number quoted, are bacterial colonies recovered on SCDB plates after 24 h incubation at 37 °C and are means of duplicate experiments).

Adherence of bacterial variants to coated and non-coated polystyrene sheets is depicted in Figure 1.

 Among Gram-negative bacteria, *E. coli *had the least adherent activity to polystyrene surface having the colony count of just 26, at time zero while *P. aeruginosa *was the most adherent to the silver coated polymers with 924 colony counts. The numbers of adhered bacteria were reduced when incubation time was increased from 40 to 80 min. The coated polystyrene sheets were most effective against *E. coli *and *S. epidermidis *after 80 min by reducing the number of bacteria from 121 to 0 and from 531 to 3 for *E. coli *and *S. epidermidis*, respectively. 

The antibacterial effects of silver coated polymers were primarily evaluated using the method stated in section microbial analysis. All the bacteria tested were inhibited by silver coated polymers while tubes containing non-coated polymers were turned turbid indicating bacterial growth.

The activity of silver coated polymers against the tested bacteria was verified using agar diffusion methods. Polymer sheets with thickness range 20 - 300 nm silver showed similar inhibition zone. This indicates that silver release by the coated sheets is limited and thickness of silver might well guarantee longer silver ion release by the surface of polymers.

Paper discs loaded with 0.5, 1, 2, 5, 10 and 20 μg of silver ion had inhibition zones of 0.9, 1, 1.2, 1.5, 1.6 and 1.7 mm respectively on the SCDB plates containing *P. aeruginosa. *The silver coated polymer discs showed inhibition zone of 1.1 mm. It is therefore estimated that the load of silver ion released from polymer discs corresponds to 1-2 μg. Considering the surface correction coefficient, the quantity of silver ion release from polymer surface was calculated as 1.57-3.14 μg/cm^3^. 

*Chemical analysis*

Silver dithizone complex is purple, therefore it could modify the absorption spectrum of pure dithizone. So, it is possible to estimate silver ions presence in the sample solution based on decrease in dithizone absorbance by silver ions. 

**Figure 2:**
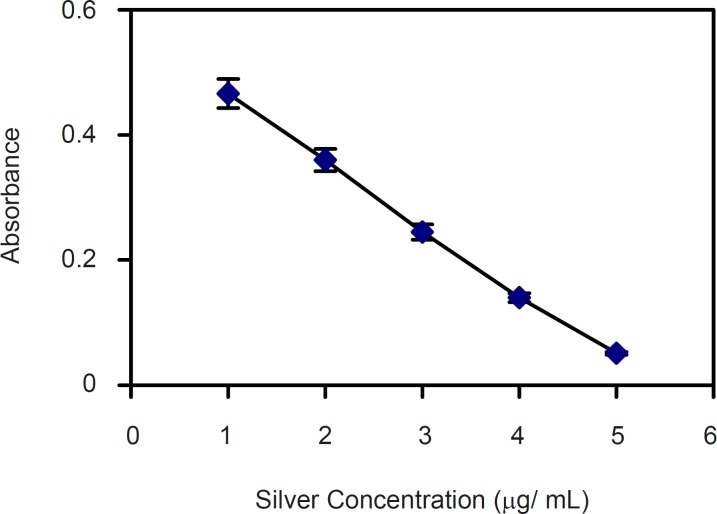
Calibration curve of silver ion concentration measured as free dithizone at 605 nm. Y=0.45/X + 0.07

For quantitative estimation of silver ion released from the polymers, primarily a calibration curve for pure silver ions complex with dithizone at 460 nm and 605 nm (λ max of silver-dithizone complex) were constructed. Silver ions concentrations were not proportional with the absorbance of silver complex with dithizone at 460 nm. Quite in contrast, as shown in Figure 2 the concentration of silver ions (1-5 μg) is inversely proportional with the absorbance of the remained dithizone at 605 nm. Therefore, the concentration of silver ions released from polymers surface was obtained at 605 nm based on the absorbance of the remained dithizone. The results showed that the release of silver ions from 1 cm^2^ of polystyrene sheet during 12 h period was 2-4 μg. 

Investigating the plating thickness and plating rate by the method at section 2.6 showed a thickness above 20 nm with good antibacterial effects. The plating rate for this electroless silver bath was 10 to 20 nm per minute. 
